# Nanomaterials based on chitosan for skin regeneration: an update

**DOI:** 10.1097/JS9.0000000000000181

**Published:** 2023-03-24

**Authors:** Ilham B. Amor, Talha B. Emran, Hadia Hemmami, Soumeia Zeghoud, Salah E. Laouini

**Affiliations:** a Department of Process Engineering and Petrochemical, Faculty of Technology, University of El Oued, El Oued, Algeria; b Laboratory of Biotechnology Biomaterials and Condensed Materials, Faculty of Technology, University of El Oued, El Oued, Algeria; c Renewable Energy Development Unit in Arid Zones (UDERZA), University of El Oued, El Oued, Algeria; d Department of Pharmacy, Faculty of Allied Health Sciences, Daffodil International University, Dhaka, Bangladesh; e Department of Pharmacy, BGC Trust University Bangladesh, Chittagong, Bangladesh

*Dear Editor*,

Highlights

- Skin is composed of several layers essential to its response and function to injury.
- Wound healing process is to achieve a quick and effective skin tissue regeneration.
- Biopolymers, such as chitosan, have been widely utilized in regenerative medicine.
- Nanomaterial topical use has been shown to dramatically enhance skin tissue healing.

Skin is composed of several layers essential to its response and function to injury: hypodermis, dermis, and epidermis. Damaged skin tissue spontaneously starts the wound healing process when injured^1^. Inflammation, hemostasis, remodeling, and proliferation (or scar tissue development) stages make up the spontaneous and dynamic repair process that occurs in the wounded tissue during wound healing. Hemostasis is the initial stage following skin injuries, and bleeding is one of the main reasons for early demise^2^. By replacing tissue that has been destroyed and repairing damaged tissue, wound healing attempts to mend the damaged tissue^3^. High cellularity characterizes the inflammatory phase (1–3 days after injury), which brought on by the migration of macrophages, neutrophils, and lymphocytes to the site of the wound. This phase functions to clean the wound bed using attracting inflammatory cells to the wound site and promoting hemostasis. The next stage of healing of wounds is the proliferative granulation tissue formation phase (4–14 days after wounding). This stage is characterized by the growth of fibroblasts at the site of the wound and the production of crucial extracellular matrix (ECM) such collagen, elastin, and glycosaminoglycans. The last stage of wound healing is known as remodeling (or scar tissue development), and it is characterized using the replacement of immature type 3 collagen with mature type 1 collagen. As the tissue gets less vascularized in this stage, the skin tissue healing process is ultimately complete^4^.

The goal of the wound healing process is to achieve a quick and effective skin tissue regeneration with maximum function, little to no scarring, and no keloid development. Successful skin tissue healing, however, continues to be a biological challenge and a significant healthcare concern. Especially persistent wounds, such as diabetic and ischemic wounds, often lead to functional ability loss and a rise in pain and infections^4^. To encourage complete skin tissue healing with the fewest possible adverse effects and expenses, innovative and effective treatments are now being developed vigorously^5^. To do this, biopolymers, such as chitosan, have been widely utilized in regenerative medicine. Tissue regeneration is accomplished using a variety of adaptable biomaterials that are used to repair, maintain, or enhance damaged skin tissues.

Chitosan-based nanomaterials have made great help for the creation of new, appropriate materials for use in a there are numerous application in biomedicine, including those for biosensors, drug delivery, tissue engineering, and diagnostics^6^. Nanoparticles (NPs), nanogels, and nanofibrous scaffolds are the most prevalent types of chitosan-based nanomaterials. Nanomaterials have significant benefits related to their nanometer sizes, such as their very large surface area compared with their volume and the capacity to enter into cytoplasmic space (increased cellular uptake). Therapeutic substances might be added to chitosan NPs to increase their bioavailability, chemical stability, and biocompatibility, while allowing the pharmacokinetics and pharmacodynamics of the therapeutic agent to be modified, this increases the active drugs’ effectiveness and decreases their toxicity^7^–^9^.

Nanomaterial topical use has been shown to dramatically enhance skin tissue healing in tissue engineering^10^. This results from interactions between several biomolecules and ECM elements at the nanoscale^11^. The primary biological element of the ECM is collagen, which contains fibers that range in size between 50 and 500 nm. Chitosan-nano-based materials, in particular, chitosan nanofibers releasing active drugs, are considered suitable scaffolds for skin tissue engineering^12^.

Since biological cells cannot recreate their original structure without an extracellular guide (scaffold material), the existence of a scaffold is essential for the effective restoration of the original tissue architecture^13^. Interestingly, the phenotype of cells growing on nanofibrous scaffolds resembles that of cells growing in their native environment. By comparing the nanostructure of nanofibrous materials to that of natural ECM components (collagen), this phenomenon may be understood. Because nanofibrous materials have the ability to direct native human cells along their surface, healing the damaged region, topical administration of these materials can be employed for skin tissue regeneration^13^. Thus, chitosan-based fibrous nanostructures have a tremendous potential to encourage skin regeneration by resembling the milieu in which human cells and tissues are found naturally. In addition, compounds for the prolonged release of medicinal medicines and biological substances including DNA and proteins have been developed using chitosan-based nanofibrous materials^6^.

There are several methods to create three-dimensional nanofibers, such as electrospinning, phase separation, self-assembly, drawing, and template synthesis^6^. The most often used method is electrospinning since it is effective and easy. It may be used to create nanofibers with diameters between 5 and 500 nm^14^. The method is based on injecting a polymer solution into a syringe, then expelling the polymer solution via a metal capillary while using a high-voltage power source^14^. Nanofibrous materials cannot be formed from aqueous solutions of pure chitosan using the electrospinning technique^15^. As a result, chitosan is modified or combined with other synthetic and natural polymers for use in tissue engineering, medication delivery, and healing of wounds^16^. Recently, Zhao *et al.* 10 reviewed the synthesis of nanofibers with renewable polymers, including chitosan. In their review article, the authors highlighted the harmful aspects of preparing chitosan nanofibers in organic solvents for biomedical applications. To enhance the mechanical properties of the nanofibers, the majority of chitosan-based nanofibrous materials combine chitosan with other polymers in an aqueous solution. Due to their biodegradability, biocompatibility, antibacterial, hemostatic, and other properties, chitosan-based nanofibrous materials can be successfully employed for skin regeneration, tissue engineering, wound healing, and hemostasis depending on their chemical composition, and mechanical characteristics, as well as their capacity to allow cell attachment and growth on the surface of nanofibers^10^.

Chitosan-based NPs have furthermore been used for wound healing to chitosan-based nanofibrous scaffolds for the continuous release of therapeutic agents (such as growth factors or antibiotics) in skin tissue regeneration^17^. As was already mentioned, chitosan itself promotes wound healing. The combination of chitosan with medicinal substances in the form of chitosan-based NPs may improve this effect. Reverse micellization^18^, reverse emulsion^19^, desolvation^20^, emulsion solvent diffusion^21^, and electrostatic complexation are the techniques utilized to create chitosan NPs^22^. The simplest of these processes, electrostatic complexation, produces NPs with repeatable size distributions and is carried out in an aqueous solution at ambient temperature and atmospheric pressure. Chitosan NPs can be created when protonated amino groups of chitosan combine with anionic macromolecules such hyaluronic acid, alginate, and DNA or with molecules like (poly) phosphate, sulfate, and citrate to form complexes. This is the last feature characteristics (nontoxicity, hemocompatibility, mucoadhesion, and antibacterial activity), in addition to their capacity to effectively integrate therapeutic compounds, that make them acceptable for biomedical applications^23^. Chitosan NPs may integrate hydrophilic, hydrophobic, and macromolecules, resulting in a prolonged release of the medications they are encapsulating. This method has been applied in a number of biological settings, including the regeneration of skin tissue.

Nanogels are a significant subset of chitosan nanomaterials. Hydrogels in the nano-dimensional range are referred to as nanogels^17^. Changes in pH, temperature, and ionic content can cause certain nanogels to become stimulus-responsive, which is crucial for drug administration in topical applications^17^. Nanoscale cross-linked networks of the same or distinct kinds of polymers make up nanogels, which have a high water absorption capacity^10^. Due to the presence of hydrophilic groups on polymer chains, certain nanogels (hydrogels) can expand when exposed to water^10^. Nanogels with water-absorbent properties have structures like those of human tissues, making them appropriate for tissue engineering. Therapeutic drugs, biomacromolecules (growth factors, proteins), and NPs can all be included inside the cross-linked network of nanogels^24^. The persistent and localized release of the therapeutic substance to the target region is made possible by the polymeric structure of the nanogels, which shields the entrapped molecules from quick dissolution^25^. Depending on the kind of connections connecting various macromolecular chains, nanogels can be categorized as either permanent or reversible. The nanogel is referred to as reversible when secondary forces, like H-bonding and ionic or hydrophobic forces, play the primary role in building the nanogel network. Permanent networks are those created by covalent linkages as a result of polymer cross-linking^26^. Therefore, novel materials with beneficial qualities for skin regeneration may be created using a variety of nanogel production techniques. As scaffolding for cell adhesion, nanogels have been widely employed in regenerative medicine to enable cellular remodeling that replicates the normal cellular environment^25^. Nanogels have been used in surgical coatings, prosthetic skin, cartilage, and corneas in this approach. The oral, nasal, ocular, topical, vaginal, and subcutaneous routes are all acceptable ways to give nanogels^25^. As a result, nanostructured chitosan can be generated as NPs, nanofibers, or nanogels that are appropriate for skin regeneration.

## Provenance and peer review

Not commissioned, internally peer-reviewed.

## Ethical approval

Not applicable.

## Sources of funding

None.

## Authors’ contribution

I.B.A.: conceptualization, data curation, writing – original draft preparation, writing – reviewing and editing. T.B.E.: writing – reviewing and editing, visualization, supervision. H.H., S.Z., and S.E.L.: data curation, writing – original draft preparation, writing – reviewing and editing.

## Conflicts of interest disclosure

The authors declare that they have no financial conflict of interest with regard to the content of this report.

## Research registration unique identifying number (UIN)

Not applicable.

## Guarantor

Talha B. Emran.
